# Primary malignant pericardial mesothelioma presenting with cardiac tamponade

**DOI:** 10.1016/j.ijscr.2020.07.054

**Published:** 2020-07-17

**Authors:** Shigefumi Matsuyama, Tomohiro Imazuru, Masateru Uchiyama, Hiroo Ota, Mitsuru Iida, Tomoki Shimokawa

**Affiliations:** a Department of Cardiovascular Surgery, Toranomon Hospital, 2-2-2 Toranomon, Minato-ku, Tokyo, 105-8470, Japan; b Department of Cardiovascular Surgery, Teikyo University Hospital, 2-11-1 Kaga, Itabashi-ku, Tokyo, 173-8606, Japan

**Keywords:** Primary malignant pericardial mesothelioma, Cardiac tamponade, Surgical intervention

## Abstract

**Introduction:**

Primary malignant pericardial mesothelioma is a rare tumor that is very difficult to diagnose. Furthermore, it is a lethal disease, because patients usually have progressed at the time of referral.

**Presentation of the case:**

We report a 44-year-old man with primary malignant pericardial mesothelioma. He was referred to our hospital for the diagnosis and treatment of a massive pericardial effusion and huge tumor. Pericardiocentesis was performed, but we could not obtain definitive diagnosis, and the cardiac tamponade continued along with the signs/symptoms. He required surgical intervention for the diagnosis and treatment. After surgery, his signs/symptoms improved. He received adjuvant therapy, although he died 7 months after surgery.

**Discussion:**

Primary malignant pericardial mesothelioma is a rare tumor. The most common signs and symptoms are related to constriction of the heart by the tumor and/or effusion. Even if the pericardial fluid specimen obtained by pericardiocentesis is negative for malignant cells, primary malignant pericardial mesothelioma should be included in the differential diagnosis. Because the malignancy is usually advanced at the time of diagnosis, it has been difficult to cure. Radiation and chemotherapy have been used in addition to surgery, but have been minimally effective.

**Conclusion:**

The outcome of our patient with pericardial malignant mesothelioma was dismal. The indications for surgical intervention should be carefully considered except for critical cases requiring alleviation of immediate life-threating conditions.

## Highlights

• Primary malignant pericardial mesothelioma is rare, and its diagnosis is difficult.
• The surgical intervention was performed after pericardiocentesis for definitive diagnosis and treatment.
• The prognosis of malignant pericardial mesothelioma was very poor.
• The indications for surgical intervention should be carefully considered except for critical cases.

## 1 Introduction

Primary malignant pericardial mesothelioma (PMPM) is a rare tumor that is very difficult to diagnose. The treatment options are limited because of its rapid growth. Surgical treatment can be curative if the tumor is completely resected [1,2]. However, because patients with PMPM frequently present late in the disease, surgical intervention is usually palliative. In this report, we describe in line with the SCARE criteria [3] a 44-year-old man who presented with cardiac tamponade and pericardial tumor.

## 2 Case report

**Fig. 1:**
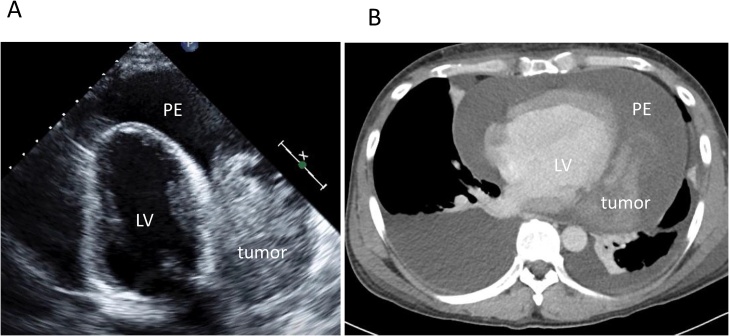
A. Transthoracic echocardiography findings. B. Computed tomography findings. Massive pericardial effusion and large tumor were detected. PE: pericardial effusion, LV: left ventricle.

**Fig. 2:**
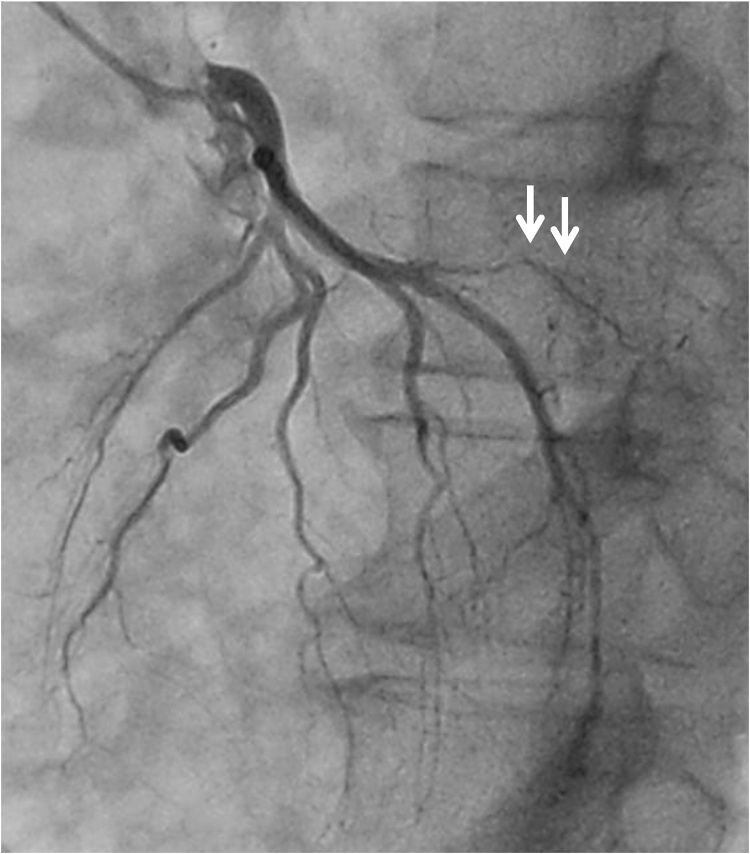
Preoperative coronary arteriography findings. Feeding vessel of the tumor extends from the circumflex branch (white arrows).

**Fig. 3:**
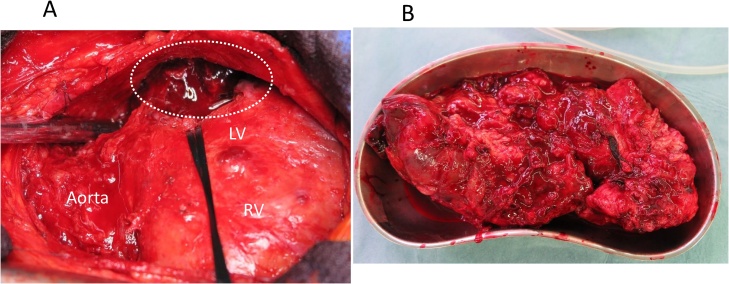
A. Operative findings. The large tumor occupies the lateral to posterior pericardial space (dotted circle). LV: Left ventricle, RV: Right ventricle. B. Resected tumor.

**Fig. 4:**
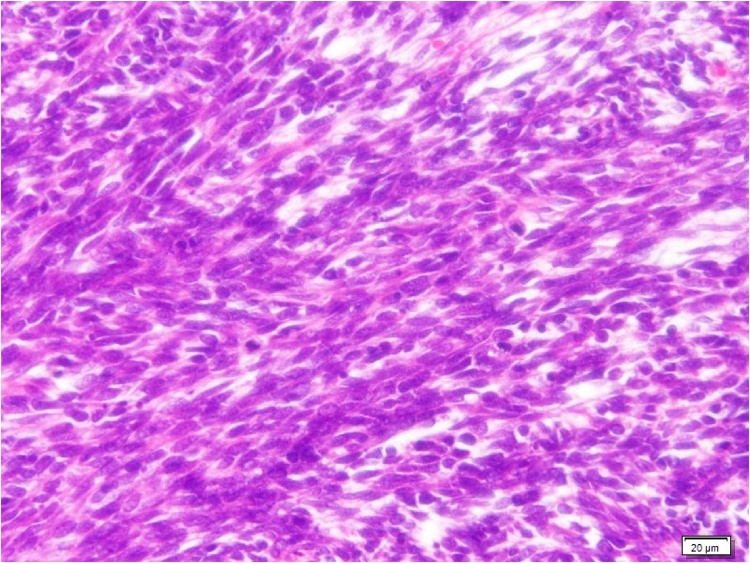
Histopathological findings of the resected tumor. The histopathological examination showed dense proliferation of spindle cells with hemorrhage and necrosis.

**Fig. 5:**
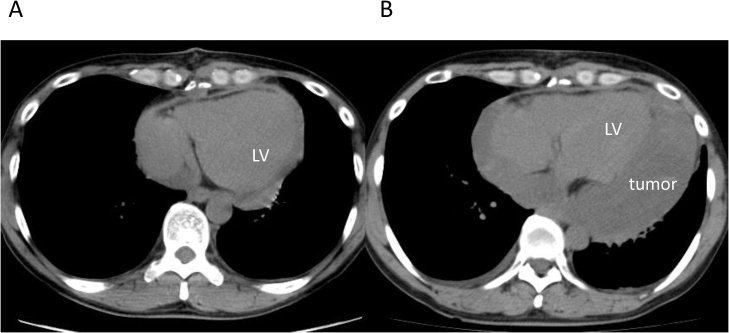
A. Computed tomography findings from postoperative day 10. The mass is almost gone. B. The findings of computed tomography 2.5 months after surgery. The regrown mass occupies the pericardial space and compresses the left ventricle (LV).

The patient was a 44-year-old man who had gone to a regional hospital with worsening dyspnea on exertion and lower extremity edema approximately 2 months prior to admission. He was referred to our hospital for diagnosis and treatment. The patient did not have an obvious history of occupational or incidental exposure to asbestos. The physical examination at admission revealed jugular distension and lower extremity edema. His blood pressure was 120/58 mm Hg and heart rate was 120 beats/min. Transthoracic echocardiography and computed tomography showed massive pericardial effusion, with a maximum diameter of 53 mm at the apex of the pericardial sac and a large tumor (95 × 99 mm in diameter) occupying the lateral to posterior pericardial space (Fig. 1A, B). The tumor showed heterogeneous contrast enhancement, and its border was unclear. Computed tomography showed lymphadenopathy of the mediastinal and subcarinal lymph nodes. Coronary angiography revealed that feeding vessels of the tumor extended from the circumflex artery (Fig. 2). Thoracentesis and pericardiocentesis were performed; however the signs/symptoms of tamponade remained. Cytological findings of the pericardial fluid specimen was grade II and was negative for malignant cells. After a preoperative discussion, our team decided upon a surgical intervention for the definitive diagnosis and to reduce the tumor mass as much as possible to improve the patient’s symptoms. A median sternotomy and pericardiotomy were performed, and the bloody pericardial effusion was removed. The pericardium was thickened. The large tumor was adherent to the epicardium and pericardium and extended from the lateral to posterior side (Fig. 3A). We performed a blunt dissection of the adhesions. The margins between the tumor and epicardium were unclear and most of the tumor was bluntly dissected. Some of the pericardium with adhesions to firm tumor tissue was removed, and the opened pericardium was not repaired so that pericardial fluid could drain to the thoracic space to alleviate cardiac tamponade. Only the tissue that included the feeding vessels was ligated and cut with an Endo GIA surgical stapler (Medtronic, Minneapolis, Minnesota, United States) in order to cut and ligate safely. Cardiopulmonary bypass was not needed for removal of the large tumor, which was resected as much as possible. We resected the large tumor by dividing it into sections. The weight of the resected tumor was 480 g (Fig. 3B). The histopathological diagnosis of the resected tumor specimen was malignant mesothelioma, sarcomatoid type (Fig. 4). The pericardial fluid obtained at surgery showed grade V cytology. It showed irregularly shaped nuclei and numerous mitotic divisions, and the cells were identified as malignant cells. The patient’s symptoms improved and his early postoperative course was uneventful. Postoperative computed tomography showed reduction of the tumor mass (Fig. 5A). We recommended adjuvant therapy for the patient because of his young age and he and his family agreed to proceed. At 3 weeks after surgery, he was administered carboplatin and pemetrexed; however, the tumor progressed. The tumor was irradiated, with a temporary reduction in size. However, 2.5 months after surgery, the tumor regrew and compressed the left ventricle (Fig. 5B). Heart failure developed. Chemotherapy was discontinued because of hematotoxicity. Despite maximum radiotherapy, the tumor started to increase in size. The patient was transferred to hospice care for palliative therapy and died 7 months after surgery.

## 3 Discussion

PMPM is rare. Its prevalence in a large autopsy study was less than 0.0022% [4], although it is one of the common pericardial tumors in some clinical series [5]. McGehee and colleagues recently provided review of 103 published cases [6].

The relationship between PMPM and asbestos exposure is unclear [1,7]. PMPM is difficult to diagnose; patients with PMPM have frequently been referred for work up of pericarditis and cardiac tamponade. The most frequent signs and symptoms are related to constriction of the heart by the tumor and/or effusion. Our patient was referred to our hospital because of massive pericardial effusion and pericardial tumor. Pericardiocentesis was performed without alleviation of the patient’s signs/symptoms. The pericardial fluid showed grade II cytology. We performed a surgical intervention for the definitive diagnosis and to relieve the patient’s symptoms and cardiac constriction. The cytology of the surgical specimen of pericardial fluid was grade V. Even if the pericardial fluid specimen obtained by pericardiocentesis is negative for malignant cells, PMPM should be included in the differential diagnosis. Only 10%–30% of cases have been diagnosed by pericardiocentesis [1,6,7]; the definitive diagnosis of PMPM has mostly been obtained from specimens obtained by surgery [1,2,[4], [5], [6], [7], [8], [9], [10]].

According to the World Health Organization classification, there are 3 histological types of malignant mesothelioma, as follows: epithelioid, sarcomatoid, and biphasic [11]. Epithelioid type mesothelioma is the most frequent, and sarcomatoid type, which was the classification of our patient’s tumor, is the least frequent [7]. Because PMPM is rare, the clinical features of the 3 types are unknown. Metastatic disease has been reported in 30%–50% of patients, with the most frequent site being the lymph nodes [2,7]. In our patient, positron emission tomography-computed tomography showed increased radiotracer uptake in the mediastinal and subcarinal lymph nodes. Metastatic lymph nodes have been reported to be a predictor of decreased survival [6].

Complete resection of the tumor is the only curative treatment [1,2]. Because PMPM has usually been advanced at diagnosis, it has been difficult to cure. Surgical treatment has been reported to consist of resection of the tumor with pericardiectomy and pericardial window placement [1,2,4,[7], [8], [9], [10]]. During the preoperative discussion among our team, we predicted that the tumor was malignant and that the patient’s prognosis was poor, but we thought that it was essential to improve his symptoms. We concluded that it would be better for the patient if we performed a biopsy and surgical mass reduction as soon as possible during the same procedure. To achieve adequate reduction of the tumor mass and ligate the feeding vessels safely, we performed the surgical intervention through a median sternotomy. Radiation and chemotherapy have been used in addition to surgery, but have been minimally effective [1,2,4]. McGehee and colleagues reported at survival benefit for chemotherapy using platinum agent [6]. Adjuvant therapy that included carboplatin was administered to our patient, with radiation showing temporary efficacy, followed by regrowth of the tumor. The patient was ultimately transferred to hospice care for palliative therapy. Aigner and co-workers reported on the survival benefit of a new therapy for malignant pleural mesothelioma that uses isolated thoracic perfusion with chemofiltration [12]. We hope to perform a new trial for PMPM.

The survival of patients with PMPM has been very poor. The mean or median survival time from the first disease manifestations has been reported to range from 3.5 to 9 months [1,6,7]. Our patient died 9 months after the first manifestations. Because our patient was found to have metastatic lymph nodes preoperatively, we predicted a dismal outcomes.

In summary, because the prognosis of PMPM is very poor, the indications for surgical treatment should be carefully considered, except for critical cases presenting with pericardial constriction or suppression of cardiac function by the tumor.

## Conflicts of interest

No authors have any conflict of interest.

## Funding

No funding was received

## Ethical approval

Ethical approval was not required for this case report in our institution.

## Consent

The patient provided written, informed consent to the publication of this case report.

## Author contribution

Dr. Shigefumi Matsuyama ― corresponding author; reviewing patient notes, writing articles, analysing images, approving final submission.

Dr. Masateru Uchiyama ― revising article and collecting the data and images.

Dr. Hiroo Ota ― carrying out the research and collecting the data and images.

Dr. Mitsuru Iida ― revising article and collecting the data and images.

Dr. Tomohiro Imazuru ― revising article and collecting the data and images.

Dr. Tomoki Shimokawa ― major contributing in writing the article and approving final submission.

## Registration of research studies

Not applicable (This report is not clinical trial).

## Guarantor

Dr. Shigefumi Matsuyama.

## Provenance and peer review

Not commissioned, externally peer-reviewed.
